# Retraction: Lysyl Oxidase Polymorphisms and Susceptibility to Osteosarcoma

**DOI:** 10.1371/journal.pone.0275707

**Published:** 2022-09-29

**Authors:** The PLOS ONE Editors

After an internal investigation of this article [1], the *PLOS ONE* Editors have reason to believe it was published following a compromised peer review process. The staff editors and the handling editor did not identify these concerns prior to publication.

In light of these concerns, the *PLOS ONE* Editors retract this article.

HS did not agree with retraction. CH responded but expressed neither agreement nor disagreement with the editorial decision. YL, BL, WY, ZH, GS, RG, CW, LY and YZ either did not respond directly or could not be reached.
